# Pregnancy Care for Patients With Super Morbid Obesity

**DOI:** 10.3389/fped.2022.839377

**Published:** 2022-07-19

**Authors:** Kelsey Olerich, David Soper, Shani Delaney, Mary Sterrett

**Affiliations:** ^1^Department of Obstetrics and Gynecology, University of Washington, Seattle, WA, United States; ^2^Department of Obstetrics and Gynecology, Medical University of South Carolina, Charleston, SC, United States

**Keywords:** obesity, morbid, pregnancy, delivery, obstetric, body mass index

## Abstract

The patient with obesity represents unique challenges to the medical community and, in the setting of pregnancy, additional risks to both mother and fetus. This document will focus on the risks and considerations needed to care for the women with obesity and her fetus during the antepartum, intrapartum, and immediate postpartum stages of pregnancy. Specific attention will be given to pregnancy in the setting of class III and super morbid obesity.

## Introduction

Being overweight or obese is a common comorbidity, and the prevalence is increasing (1). Recent estimates suggest that over 66% of adults in the United States are overweight, as indicated by a BMI higher than 25 kg/m^2^ (2). In the United States, over 30% of reproductively-aged women are obese and obesity is one of the most significant contributors to overall health (2, 3). The prevalence of obesity in general, and particularly super obesity, is increasing in our obstetric population (4). Data surrounding the management of the obese parturient is limited to small prospective studies and expert opinion; identifying evidence-based practices for the patient with obesity is important for optimal care (5). Where evidence is lacking, we offer recommendations based on our institutional practice.

Obesity is a risk factor for many obstetrical complications including diabetes, hypertensive disorders of pregnancy, birth defects, macrosomia, preterm delivery, fetal death in utero, increased rate of cesarean delivery and anesthetic complications. The rising rate of obesity is thought to underlie the increasing incidence of type 2 diabetes in pregnancy and the antepartum sequalae of the disease. Additionally, rates of chronic hypertension are increasing, mirroring the rise in obesity (3).

BMI at or above 30 kg/m^2^ is considered obese with class I obesity defined as BMI ≥ 30 to <35 kg/m^2^, class II obesity as BMI ≥35 to <40 kg/m^2^ and class III obesity as BMI ≥40 kg/m^2^ (6). The 2015–2016 National Health and Nutrition Examination Survey (NHANES) data indicated that 36.5% of women aged 20–39 meet criteria for obesity (1). A BMI at or above 50 kg/m^2^ is considered super-obesity, and 2% of pregnant women in the United States are classified as having super-obesity (4). For the purpose of this document, the definition of the bariatric patient is any individual whose weight and/or body habitus interferes with the ability to provide safe, reasonable care. This includes any person with a body weight >300 pounds, a BMI ≥ 40 kg/m^2^ (class III obesity), or persons overweight by >100 pounds (7).

## Antepartum Care

### Maternal

#### Cardiovascular

The risk of hypertensive diseases of pregnancy, most notably preeclampsia, is increased in mothers with obesity with some studies citing a 3–10 fold increase in preeclampsia risk compared to normal weight women (8). The increased preeclampsia risk appears to exhibit a dose-response relationship with one study finding that the risk for preeclampsia doubles with each 5 kg/m^2^ increase in BMI (9). This finding appears to be particularly true for women with super-obesity who, when combined with a high rate of gestational weight gain, have been found to be at the greatest risk for development of preeclampsia (OR 7.52, 95% CI 2.7–21.0) (10). However, patients with obesity do not appear to be at a significantly elevated risk of severely preterm (<34 weeks) preeclampsia (10).

Maternal serum leptin, which is released by adipose tissue and to a lesser degree the placenta, is correlated with BMI, both during pregnancy, and in the postpartum period (11). Elevated leptin levels are associated with placental ischemia and endothelial dysfunction (11), which likely underlies its association with the development of preeclampsia (12). Maternal obesity increases the risk of peripartum cardiomyopathy; leptin contributes to vascular dysfunction and is hypothesized to contribute to peripartum cardiomyopathy in mothers with obesity. In mothers with obesity with peripartum cardiomyopathy there is evidence of decreased cardiac recovery, and instead a transition to chronic nonischemic cardiomyopathy compared to their normal weight counterparts (12).

We recommend obtaining baseline labs (CBC, CMP, LDH) and urine protein creatinine ratios in all pregnant women with obesity, regardless of the presence of chronic hypertension. Additionally, we recommend considering a baseline transthoracic echo in women with class III and super-obesity secondary to their elevated risk of cardiomyopathy and underlying vascular disease.

There are additional logistical issues, such as obtaining consistent blood pressure monitoring. This can be difficult in the upper extremities, and writing nursing notes to ensure clear locations for blood pressure sites (i.e., upper arm, wrist, size of cuff) will improve consistency and assist in clinical decisions (13). Indirect blood pressure measurements have been found to be reliable with a correctly fitting cuff, even with a large upper arm circumference (14). The American Heart Association has published an open access document that outlines how to select optimal blood pressure cuff size and optimize accurate readings (15). Invasive monitoring, such as arterial lines, is also occasionally required, though is generally outside the scope of a Labor and Delivery unit.

#### Respiratory

The elevated intra-abdominal pressure and reductions in lung volumes that are common in pregnancy are accentuated by obesity (16, 17). The incidence of obstructive sleep apnea (OSA) is also greater within the obese, and particularly super-obese, population (16, 18). In a study evaluating the diagnosis of OSA in patients being screened for weight loss surgery, the prevalence of OSA in the super-obese group (BMI 50–59.9 kg/m^2^) was 77% (19). In those with a BMI of 60 kg/m^2^ or greater, the prevalence rose to 95% (19). Given the high prevalence of OSA in patients with obesity, as well as the significant morbidity associated with the presence of OSA in pregnancy, we recommend that all women with class III or super-obesity be screened for OSA prior to pregnancy. Screening can be performed with the STOP-bang questionnaire, as supported by the Society of Anesthesia and Sleep Medicine (20). Women with class I or II obesity, as well as normal weight women, should be screened for OSA based on the presence of OSA symptoms. Although there is a lack of data to indicate if treatment of OSA in pregnancy modifies adverse pregnancy outcomes associated with OSA (such as preeclampsia), identifying it as a comorbidity is an important risk factor for anesthesia related deaths (21).

Pregnant patients with OSA, regardless of BMI, are more likely to be diagnosed with preeclampsia (adjusted OR, 2.5; 95% CI, 2.2–2.9), eclampsia (adjusted OR, 5.4; 95% CI, 3.3–8.9), cardiomyopathy (adjusted OR, 9.0; 95% CI, 7.5–10.9), pulmonary embolism (adjusted OR, 4.5; 95% CI, 2.3–8.9), and in-hospital mortality (adjusted OR, 5.28; 95% CI, 2.45–11.53) (22). However, there is limited data on if treatment of OSA improves outcomes (4).

#### Endocrinology

Pregnancy is an insulin resistant state and may unmask previously subclinical metabolic dysfunction. Women with pregestational type 2 diabetes are urged to obtain tight control prior to attempting pregnancy and should seek care with a Maternal Fetal Medicine specialist early in pregnancy.

Maternal obesity is also a significant risk factor for the development of gestational diabetes. The degree of maternal obesity and incidence of gestational diabetes are linearly correlated with class I obesity affording a RR of 2.94 (2.73–3.18) while the presence of class III obesity affords a RR of 3.55 (3.26–3.86) compared to normal weight women (8, 23).

Vitamin D deficiency is more common in the general population with obesity and pre-pregnancy obesity is associated with both maternal and neonatal vitamin D deficiency (24). Vitamin D deficiency has been reported in almost 60% of pregnant women with obesity compared to ~35% of normal weight women (25). Vitamin D deficiency in pregnancy has been associated with a significantly increased risk of developing preeclampsia as well as worsening glucose tolerance, two pregnancy-related complications for which women with obesity are already at an elevated risk (24). Hypovitaminosis D is also associated with low birth weight and a possible association with increased cesarean birth rates (24).

The United States Preventative Services Task Force (USPSTF) recommends screening for gestational diabetes in asymptomatic pregnant persons at 24 weeks gestation or after, and feels that the current evidence is insufficient to screen for GDM prior to 24 weeks (26). The USPSTF recognizes that for patients with risk factors for type 2 diabetes (obesity, family history of diabetes, fetal macrosomia in a prior pregnancy) that a clinician should use their clinical judgement to determine what is appropriate screening for that patient in the first or early second trimester (26). We recommend screening at presentation to pregnancy care for gestational diabetes in all patients with obesity; if negative, we recommend repeat screening at the standard 24–28 weeks estimated gestational age.

We also recommend screening in the first trimester for vitamin D deficiency in all pregnant women with obesity, with subsequent supplementation when appropriate.

Despite the unclear mechanistic link between obesity and thyroid dysfunction, thyroid dysfunction and adverse pregnancy outcomes is well-described (27). Due to this, the American Thyroid Association recommends screening patients with a BMI ≥ 40 for thyroid dysfunction (28). Notably, ACOG does not include a BMI category as an indication for thyroid screening. Specifically, ACOG outlines that screening should be performed only in patients with a personal or family history of thyroid disease, type 1 diabetes mellitus, or clinical suspicion of thyroid disease (29). ACOG does not support universal screening due to the lack of cognitive improvement for children in those women with subclinical hypothyroidism who were treated during pregnancy (30, 31).

#### Gestational Weight Gain

In 2009, the Institute of Medicine (IOM) published guidelines regarding the amount of gestational weight gain recommended in order to optimize outcomes for patients and neonates (32). The IOM guidelines recommend a weight gain of 11–20 lbs for all patients with obesity throughout the course of their pregnancy (32). Unfortunately, secondary to limited data examining gestational weight gain by obesity class, the recommendations are not further specified for patients with higher-order obesity.

It is estimated the ~40–50% of women with obesity gain greater than the recommended amount of weight during pregnancy (3, 32). Excessive gestational weight gain is a risk factor for postpartum weight retention, which can exacerbate the metabolic dysfunction for which women with obesity are already at risk.

Multiple studies have investigated restricted gestational weight gain, below IOM recommendations (generally defined as <11 lbs/5 kg), in women with pregestational obesity, and have found an increased rate of small for gestational age (SGA) infants (aOR ranging between 1.2 and 2.6) (33–35). Some perceived benefits to limited weight gestational gain in these patients have been suggested, including decreased risk for cesarean section and decreased post-partum weight retention; however, secondary to the lack of proven benefit and the known increased risk of SGA infants, we recommend advising patients according to the IOM guidelines for gestational weight gain. The USPSTF has published a table of behavior counseling interventions to guide practitioners' discussion with pregnant patients who are at risk of adverse outcomes related to obesity (36). These recommendations include structured exercise classes, healthy eating habits, directed counseling and goal setting.

We recommend determination of BMI at a patient's first prenatal visit with targeted counseling regarding recommendations for weight gain, as well as strategies that can be employed to limit weight gain.

### Fetal

#### Pregnancy Loss

Women with obesity have an increased risk of spontaneous abortion (SAB) as well as recurrent miscarriage (3, 8, 37). The risk of SAB for women with a BMI >30 kg/m^2^ is increased by 30% (OR 1.31, 1.18–1.47)(8) and, for those with class II and III obesity, it is further increased with RR 1.97 (1.71–2.28) and 3.54 (2.56–4.89), respectively (23).

##### Fetal Anomalies and Genetic Screening

Fetal anomalies, including neural tube defects, hydrocephaly and cardiac, orofacial and limb reduction defects, are more commonly observed in women with obesity (3, 8). However, there is a decreased prevalence of gastroschisis in women with obesity (OR 0.17, 0.10–0.30) (3, 23, 38). There are various theories regarding the increased incidence of anomalies including underlying insulin resistance/hyperglycemia, nutritional deficiencies as well as difficulty in ultrasound diagnosis leading to a possible decrease in termination for fetal anomalies (3, 38–40).

Ultrasound detection of congenital anomalies is compromised in pregnant women with obesity, with significantly decreased ultrasound sensitivity for fetal structural anomalies with increasing maternal obesity class (3). Furthermore, because of the increased volume of distribution related to increased plasma volume, measurements of serum analytes can be altered, and cell free fetal DNA screening is more likely to yield an indeterminate result. While obesity should not be seen as a contraindication to diagnostic genetic testing, both chorionic villus sampling and amniocenteses can be technically challenging in patient with obesity.

In line with the American Institute of Ultrasound in Medicine guidelines (41), we recommend a detailed midtrimester anatomic ultrasound for all patients with a pre-pregnancy BMI >30 kg/m^2^. Additional techniques, such as transvaginal US or placing the US probe in the maternal umbilicus, should be employed as ways to mediate the effect of maternal obesity. In the absence of other indications for an early 2nd trimester fetal ultrasound, we recommend scheduling the fetal anatomic survey at or shortly after 20 weeks to facilitate the best chance to identify fetal structures in patients with obesity. There has been discussion regarding the use of MRI in the pregnant patient with obesity. An elevated BMI could warrant the use of MRI, which would be expected to have less impact from deeper tissue layers compared to ultrasound imaging. There is limited availability and relatively high cost associated with the use of MRI, but no expected adverse effects. Because of this, we would consider the use of MRI to assess fetal anatomy if an anomaly is suspected, but would not routinely offer MRI (42).

#### Growth Abnormalities

Pre-pregnancy obesity increases the risk for large for gestational age infants (class I obesity RR 1.74, 1.65–1.83, class III obesity 2.3., 2.14–2.52)(8) as well as fetal macrosomia (defined as birthweight >4,500 gm; OR 3.23, 2.39–4.37) (8, 23). Though it has previously been attributed to underlying comorbidities, there is recent evidence that severe fetal growth restriction (FGR) is more common in women with obesity and that umbilical artery Doppler abnormalities are more frequent with increasing obesity class (43). The inflammatory effects of obesity on the placenta and intrauterine environment are the proposed etiology for this potential association.

Due to increased inaccuracy in fundal height measurements (44), we recommend considering serial growth ultrasounds vs. a follow up growth US in the third trimester in women with obesity.

#### Stillbirth

At all gestational ages, the risk of stillbirth increases with increasing maternal obesity class (3). Though the absolute risk remains low (59/10,000 in women with BMI 30–35 kg/m^2^ compared to 40/10,000 in women with BMI 20–25 kg/m^2^) (45), a large-scale retrospective cohort study found that women with obesity had increased rates of stillbirth, compared to normal weight women, at each gestational age studied with adjusted hazard ratios ranging between 1.4 and 3.3 for women with class III obesity, depending on gestational age (46). This increased rate was particularly true at later gestational ages with women with a BMI > 50 kg/m^2^ having a 5.7-fold and 13.6-fold increased risk of stillbirth at 39 and 41 weeks, respectively, compared to normal weight women (46). ACOG has published estimated rates of stillbirth for maternal or fetal conditions, and an elevated BMI is included (47).

The mechanism for the increased stillbirth rate remains unknown; however, theories include underlying undiagnosed comorbidities, chronic intrauterine inflammation, hypoxia, placental dysfunction (45, 48). Recent ACOG guidance recommends considering antenatal testing at 37 weeks for pregnancies complicated by pre-pregnancy BMI of 35–39 kg/m^2^, and consider starting at 34 weeks if pre-pregnancy BMI was ≥40 kg/m^2^ (3). We concur with the ACOG antenatal testing considerations, or as dictated by comorbid conditions or at the discretion of the provider.

#### Preterm Delivery

Obesity is associated with an increased rate of preterm delivery (8, 23); however, the distinction between spontaneous and iatrogenic preterm births has not always been made. A study conducted with both Swedish and American cohorts demonstrated that obesity is a strong independent risk factor for extremely early (22–27 weeks EGA) spontaneous preterm delivery (class III obesity RR 2.21, 1.76–2.77). However, obesity was found to have no effect on spontaneous preterm delivery at more mature gestational ages (49).

## Intrapartum

For women with obesity, and particularly those with super-obesity, a multidisciplinary approach to their intrapartum care is required; for patients with a BMI >40 kg/m^2^, consultation with specialists should be considered; for those patients with a BMI >50 kg/m^2^, it is recommended. A multi-disciplinary care team should include Maternal-Fetal Medicine (MFM) specialists, Anesthesia, nursing and pharmacy, with potential consultation with Critical Care, Cardiology and Pulmonology colleagues.

### General Needs

#### Facility Logistics

For the bariatric patient, physical space (i.e., wider doorways, elevators with increased maximal weight allowances) and accommodating equipment designed for patients with obesity and staff trained in bariatric patient transfer is essential. Obtaining a bariatric bed and surgical table is necessary, and pre-emptively placing a hovermat on the bed will assist with any future transfer if the patient is for surgical delivery. Other considerations include portable or ceiling-mounted lifting equipment, bariatric wheelchairs and commodes, large surgical safety belts and sequential compression devices.

#### Anesthetic Considerations

Obstetric anesthesia specialists would ideally meet with the patient prior to hospitalization to evaluate comorbidities, assess pulmonary and cardiovascular status, and obtain an airway exam. This is also an opportunity to elicit history of prior difficult neuraxial placement or difficult airway. Consultation should also include a discussion of labor analgesic options including labor epidurals and anesthetic options for cesarean delivery. When discussing labor epidurals, it is important to emphasize that placement can be more difficult in obesity and can take longer to place and are more likely to need replacement (50), for that reason early labor epidural placement should be considered. Especially in cases where the patient proceeds with vaginal delivery, having early epidural placement, 24-hour access to anesthesiologists, additional staff to facilitate transfer, and fiber optic equipment for possible urgent intubation is recommended (4, 51). Early communication regarding change in status and anesthesia needs is imperative for patient care.

In the super-obese subpopulation, the incidence of failed regional anesthesia placement is 12–17% with a risk of general anesthesia ranging between 6 and 12% (52). Should cesarean delivery be indicated, additional anesthetic concerns include positioning, intraoperative blood pressure monitoring and postoperative analgesia. Positioning during cesarean delivery can be difficult and should include all team members when possible. Depending on the patient's airway exam, the anesthesia team may wish to position the patient on a ramp in case endotracheal intubation is needed. Reliable non-invasive blood pressure monitoring can be difficult in the population with obesity. Cesarean delivery can be associated with hemodynamic instability and massive hemorrhage, therefore invasive blood pressure monitoring can be considered if non-invasive techniques have proven unreliable (53). Another consideration is post-cesarean analgesia, where the gold standard in the United States for non-obese women is neuraxial morphine. There is an increased risk of hypoventilation in the setting of obesity and opioid pain medications, and collaboration with anesthetic providers is necessary for safe management. Ideally, pain control options should be discussed during prenatal care, but each case needs individual management regardless of when a patient presents for delivery.

IV access is a necessary part of labor and delivery and must be approached with the intent of obtaining access with minimal discomfort to the patient. Multiple attempts to start an IV should prompt the request for additional resources, including ultrasound guided placement, and consideration for peripherally inserted central catheters. Given the higher risk of postpartum hemorrhage and difficulty of IV placement in obesity, early placement of two peripheral IVs should be considered.

#### Delivery Approach and Labor Considerations

Obesity is an independent risk factor for cesarean delivery. Among nulliparous women, class I obesity afforded a RR of 2.26 (2.0–2.51) for a cesarean delivery while women with class II –III obesity had a RR of 3.38 (2.49–4.57) (8). Up to 50% of women with super-obesity undergo a cesarean delivery, compared to 33% of women with BMI's 30–39.9 kg/m^2^, and 43% with BMI 40–49.9 kg/m^2^ (4, 54). Women with obesity are more likely to have an abnormal labor curve, with overall duration of labor and progression through latent labor being significantly prolonged. This finding exhibited a dose-response with increasing obesity class (55). Furthermore, among those nulliparous women who achieve complete dilation, women with obesity are more likely to have a prolonged second stage of labor (aRR 1.65, 1.18–2.30, for >4 h) and a second stage arrest cesarean delivery (aRR 1.78, 1.34–2.34) or cesarean for fetal distress in the second stage (aRR 2.67, 1.18–3.58) (56).

#### Fetal Assessment

Ultrasound assessment for fetal presentation may be necessary, as Leopold maneuvers and vaginal exam may be inadequate to assess fetal presentation in the context of obesity. At times, fetal monitoring is not feasible when body habitus limits the ability of cardiotocography to pick up fetal heart rate. Identifying the optimal placement of a fetal monitor can require the use of an ultrasound. At times, the tissue penetration of a curvilinear probe may be inadequate, and an endovaginal probe can be utilized transabdominally, often in the umbilicus, to obtain imaging due to decreased umbilical tissue thickness (57).

Having a nurse remain at bedside to adjust the external fetal monitor and palpate for contractions is one option (58). Intermittent ultrasound of the fetal heart rate transabdominally could be performed, but is not an evidence-based recommendation, as meta-analysis of studies assessing intermittent auscultation vs. continuous monitoring often excluded high risk pregnancies (59).

In recent years there has been the development of products aimed at increasing the ability to trace fetal heart tones in women with obesity. The GE Monica Novii Wireless Patch System is one such product that has been shown to improving fetal heart tracings in women with obesity (60). Amniotomy and placement of internal monitors, including a fetal scalp electrode, may be necessary.

### Surgical Needs

#### Surgical Considerations

Obesity, and particularly super obesity, has been associated with increased rates of post-cesarean complications (4, 61, 62). A secondary analysis of a large Maternal-Fetal Medicine Unit (MFMU) Cesarean Registry found that women with a BMI >45 had a significantly increased rate of a wound complication composite, as well as each individual complication. The increased rate was most notable for wound infection (aOR 3.78, 2.60–5.46), wound opening (aOR 5.47, 2.79–10.7) and infection-related hospital readmission (aOR 2.97, 2.26–3.91) (61). The increasing risk of post-cesarean wound complications mirrors an increasing BMI, with higher complication rates seen in women with higher BMIs (62), and there is a 30–50% risk of wound complications in women with super-obesity (4).

Though less well-established than the increased post-surgical complications associated with obesity, obesity has also been found to significantly influence intraoperative characteristics and outcomes. Increasing BMI is associated with increased operative time and estimated blood loss (62). For women with super-obesity, the estimated incidence of intraoperative injury is 4–28%, and includes perioperative transfusion, reoperation, hysterectomy, bladder, bowel, or ureteral injury, or broad ligament hematomas (4).

#### Antibiotic Prophylaxis and Prep

It is recommended that all women undergoing cesarean delivery receive broad spectrum antibiotics. For women with obesity, higher doses are generally recommended. It has been demonstrated that cefazolin concentrations in adipose tissue at time of skin incision are inversely proportional to BMI when all patients are given a standard dose of 2 gm IV 30–60 min prior to skin incision (63). While all groups maintained therapeutic levels for gram positive cocci, the obese and super-obese groups were below the minimal inhibitory concentrations for gram-negative rods. Our institution has adopted guidelines for increased prophylaxis for patients with BMI >40 kg/m^2^ or weight over 120 kg. The data indicate that using 3 gm cefazolin preoperatively for these patients provides sufficient antimicrobial coverage (64). For those with a history of serious penicillin allergy, combination therapy with intravenous clindamycin 900 mg IV plus gentamicin 5 mg/kg is recommended.

Abdominal skin preparation is recommended with two or more chlorhexidine-alcohol swabs due to an increased surface area. As women with superobesity are at increased risk of postpartum endometritis, with rates estimated at 3–8% (4), vaginal preparation should be considered, especially in those women with ruptured membranes (4, 65).

#### Surgical Positioning and Incision

Operating rooms equipped with motorized lifts will make it easier to assist the patient with obesity onto the operating table (58). These rooms should have sufficient space to allow staff to move safely and efficiently (66).

Ideal surgical positioning for women with obesity, and particularly super-obesity, undergoing cesarean delivery remains unclear; however, special attention should be given to maternal pressure point reduction and pannus retraction.

The benefits of a transverse incision include increased wound strength, decreased pain and increased post-cesarean respiratory efforts; however, the infectious risk when placed under a large pannus is increased (67). Additionally, vertical incisions are typically associated with improved surgical access and visualization.

Most studies have shown that a vertical skin incision is associated with an increase in composite wound complications (including infection, seromas, hematomas, fascial dehiscence) compared to a transverse incision (61, 68, 69), as well as an increased rate of classical hysterotomy (70). A large secondary analysis of the MFMU Cesarean Registry found that, regardless of BMI, when compared to a transverse Pfannenstiel incision, vertical skin incisions were associated with increased wound complications (non-obese, 7 vs. 2%, *p* = 0.002; BMI >45 kg/m^2^, 9 vs. 5%, *p* = 0.001) (61). However, other studies have not confirmed this association (70, 71). In a study to evaluate cesarean outcomes in women with class III obesity, the increased rate of wound complications, both as a composite and individually, in those with a vertical skin incision was negated when adjusted for confounders including BMI, diabetes and pre-cesarean labor (aOR 1.7, 0.7–4.1) (70). Furthermore, vertical skin incisions were associated with less neonatal complications, including a decreased risk of 5 min Apgar scores <7 (aOR 0.06, 0.004–0.9) (70).

An additional consideration, whether the incision is transverse or vertical, is infra- or supra- umbilical incisional placement. A retrospective cohort study of parturients with a mean BMI of 49 kg/m^2^ revealed a trend toward fewer wound complications with a high-transverse skin incision compared to a low transverse incision (15.6 vs. 27.1%, *p* = 0.24); however, those in the high-transverse group had lower 5-min Apgar scores (8 vs. 9; *p* = 0.002) and increased NICU admissions (28.1 vs. 5.2%; *p* = 0.001) though no difference in umbilical artery pH levels (72).

The patient's respiratory status after pannus retraction should also help direct an accessible entry location. One option to identify placement of the skin incision is to assess the location of pannus fold origin. To do this, place one hand under the retracted pannus and one above to estimate where a skin incision would be needed to avoid exit and re-entry into the subcutaneous tissue. This assessment should occur prior to skin preparation, in order for the anticipated operative field to be adequately cleaned.

Decision regarding type of skin incision is based on the maternal body habitus distribution, access to the lower uterine segment, and maternal and physician preference; the recommended skin incision type in women with obesity has not been definitively determined (3).

#### Surgical Closure

Based on available evidence, the subcutaneous tissue should be closed in those women with at least 2 cm of adipose tissue; this approach has been shown to significantly decrease the rate of postoperative wound disruption (73). Staples or subcuticular stitches are reasonable options to consider in skin closure; a randomized control trial of women with class III obesity or above undergoing cesarean delivery found no difference in composite wound outcome between those women who received staples or those with a subcuticular skin closure (74). Though prior studies had suggested a benefit (75), a recent large multicenter randomized control trial of women with obesity undergoing cesarean sections found that negative pressure wound dressings did not decrease the risk of surgical site infections compared to standard dressings (76). Given the most recent results, we do not recommend routinely using a negative pressure dressing in all women with obesity undergoing cesarean section. A discussion with patients of the risks, benefits and cost is recommended if considering a negative pressure dressing.

## Postpartum

### General Care

Nursing care approaches for safe patient handling in the setting of obesity and super-obesity have been outlined in an online application, made available by Veteran's Affairs (7). This resource includes safe transfers, repositioning, access to abdominal and perineal areas, showering and bathing, and floor/fall recovery, amongst others. For the patient with super obesity, the postpartum period requirements may include a bariatric bed, as well as a bariatric lift to improve safety for the staff in caring for these patients. Obtaining a bariatric size bedside commode is also necessary.

For women who deliver by cesarean section there is a 1–2% risk of maternal intensive care unit admission in the super-obese population (4). Depending on the individual patient and delivery characteristics, initial post-operative recovery may necessitate an ICU setting. This is meant to facilitate increased nursing to patient ratios, invasive monitoring, and, if intubated, delayed extubation due to prolonged operative time, respiratory comorbidities, or blood loss.

Adjusted expectations for appropriate urine output based on weight and having additional resources for rapid intubation are helpful for nursing care. Once transitioned to an L&D or postpartum floor, adoption of the Maternal Early Warning Signs (MEWS) protocol is recommended (77).

Early involvement of respiratory therapy, for use of incentive spirometry or assistance with CPAP use is invaluable. Physical and occupational therapy for the patient with limited mobility is useful for those who can recover functioning to ambulate. Common immobility-related complications include skin breakdown, cardiac deconditioning, deep vein thrombosis, muscle atrophy, urinary stasis, constipation, pain management problems, and depression. Immobility also contributes to pulmonary complications such as atelectasis and pneumonia (78).

Pregnancy is a risk factor for new or persistent obesity and obesity related events in pregnancy are associated with long term metabolic dysfunction (8). Women with gestational diabetes are at increased risk for type 2 diabetes with reported rates varying widely between 2.6 and 70% when studied from 6 weeks to 28 years postpartum (79). Women with obesity are more likely to have increased development of central fat mass in the postpartum period leading to the adverse cardiovascular outcomes that are associated with central adiposity. Additionally, women with obesity have an increased risk of hypertensive diseases of pregnancy which is in turn associated with increased long-term cardiovascular risks both in the mother as well as their offspring. After a pregnancy complicated by preeclampsia, the risk of hypertension increases four-fold and the risk of stroke and ischemic heart disease increases by two-fold (80). Furthermore, the offspring of pregnancies complicated by preeclampsia are at increased risk of cardiovascular dysfunction as well. There is also an emerging body of evidence that gestational or type 2 diabetes in pregnancy as well as maternal obesity, has long-term consequences for the long-term metabolic health of their offspring (81, 82).

### Complications

#### Bleeding Risk

The increased rates of post-cesarean complications in women with obesity are discussed above. Obesity has also been associated with increased risk of postpartum hemorrhage, regardless of mode of delivery (class III obesity, RR 1.43, 1.33–1.54) (8) and providers should be aware and prepared. There has been an association of atonic hemorrhage with increasing BMI, and having uterotonics on hand for immediate or prophylactic use should be considered (83, 84).

#### VTE Prophylaxis

The incidence of venous thromboembolism (VTE) in pregnancy and the immediate postpartum period is 4–5 times higher than in the general population, and obesity further accentuates this risk with an aOR 5.3 (2.1–13.5) in women with a pre-pregnancy BMI >30 kg/m^2^ (8, 67). There also appears to be a dose-response relationship, with increasing BMI associated with increasing risk of VTE, therefore putting women with super-obesity at particularly high risk (67). Furthermore, pregnant women with a BMI >25 kg/m^2^ and a history of immobilization have an aOR of 62.3 (95% CI 11.5–337.6) for antenatal VTE, and 40.1 (95% CI 8.0–201.5) for postnatal VTE compared to women of normal BMI and no immobilization (85).

For women undergoing Cesarean birth, ACOG recommends mechanical compression devices, and pharmacologic thromboprophylaxis with additional risk factors. However, these risk factors are not specified from ACOG except for known thrombophilias or prior VTE events (86). The ACCP recommendations suggest the combined use of mechanical and pharmacologic thromboprophylaxis for patients undergoing abdominal–pelvic surgery who have Caprini scores of 5 or higher (87).

The Caprini score is a risk assessment tool to estimate the incidence of venous thromboembolism among surgical patients. In a retrospective study evaluating the Caprini score, the risk of venous thromboembolism after surgery was 0.0% for a score of 0–1, 0.7% for a score of 2, 1.0% for a score of 3–4, and 1.9% for a score of 5 or higher (88). The risk factors included in the Caprini Risk Assessment to the obstetric patient include swollen legs, BMI >25 kg/m^2^, smoking, diabetes, blood transfusions (all receiving one point), and surgery >45 min, patient confined to bed >72 h (2 points each), amongst others (89). The full table of risk factors is listed in the modified Caprini Risk Assessment Model for Pregnancy in Appendix 1 of the National Partnership for Maternal Safety- Consensus Bundle on Venous Thromboembolism or available online at https://links.lww.com/AOG/A834) (89).

For non-surgical, hospitalized patients, the Padua scoring system can be utilized, with a score of 4 or higher associated with an 11.0% risk of venous thromboembolism for patients not receiving prophylaxis, and a score of <4 with a 0.3% risk of venous thromboembolism (90).The NPMS Consensus Bundle on Venous Thromboembolism authors' interpreted the Royal College of Obstetrician and Gynaecology's (RCOG) implementation of risk based pharmacologic thromboprophylaxis as contributing to the drop in maternal mortality (89). They contended that the trend in decrease of maternal mortality followed RCOG's broader, risk-based pharmacologic thromboprophylaxis recommendations, released in 2004, for antepartum and postpartum patients. D'Alton et al. (89) utilized the Caprini and Padua scoring systems to offer antepartum and postpartum thromboprophylaxis. Specifically, these authors cited recommendations for pharmacologic thromboprophylaxis in post-Cesarean patients with additional risk factors for VTE. We recommend considering pharmacologic thromboprophylaxis for all patients who have undergone Cesarean or have a Caprini score of 5 or greater.

#### Breastfeeding

Breastfeeding has a multitude of significant health benefits both for the mother as well as the neonate. These benefits include decreased rates of obesity and diabetes in the offspring as well as increased postpartum weight-loss and decreased risk of diabetes, cardiovascular disease, hypertension, ovarian cancer and breast cancer in the mother (91). However, obesity is associated with a decreased rate of breastfeeding initiation as well as decreased duration (92). Maternal obesity has been cited as a risk factor for delayed stage II lactogenesis, which is in turn associated with breastfeeding cessation (93). It is postulated that women with obesity may have elevated baseline progesterone levels which prevent the sharp decline in progesterone after delivery of the placenta that triggers lactogenesis, and that obesity alters the prolactin response to neonatal suckling in these mothers (93, 94). Furthermore, both diabetes and cesarean deliveries have been associated with delayed lactogenesis and are more common in mothers with obesity (92).

Breastfeeding preference and behavior is multifactorial with both socio-economic and physiologic determinants. While there are many behavioral, logistic, psychologic, and cultural reasons that a woman may choose not to breastfeed, evidence suggests that obesity may make initiation and continuation of breastfeeding more difficult in women who chose to pursue it (92–94). We, as providers, should start the conversation antenatally to promote the maternal and neonatal benefits of breastfeeding, particularly in the setting of obesity. Prenatal lactation consultation can be considered to discuss the risk of delayed lactation and low milk supply. Additionally, women with obesity and super-obesity are an elevated risk for adverse delivery events including intubation and hemorrhage, which may further confound initiation of a breastfeeding relationship. Lactation consults can work with the patient and providers to determine a plan in the event of these scenarios. Together with lactation consultants, providers can help determine the appropriateness of antepartum hand expression in order to have expressed milk available for the neonate, as well as to expedite lactogenesis (95). Postpartum lactation consultation should be offered to provide additional techniques and support.

#### Contraceptive Considerations

Given the increased maternal, as well as fetal, risk associated with obesity in pregnancy, contraceptive options counseling is a vital component of preventative healthcare. Obesity can alter the pharmacokinetics of medications including contraceptives by altering the absorption, distribution and metabolism (96). However, past studies of contraceptive efficacy have routinely excluded patients who are overweight and women with obesity; therefore, inhibiting thorough and informed contraceptive counseling (96). Some studies suggest that combined hormonal contraceptives (pills/patch/ring) and progestin-only pills are less efficacious in women with obesity, though the data is mixed (96).

In addition to limited efficacy data, there is minimal safety data regarding contraceptive methods in women with obesity and no safety information specifically regarding women with super-obesity (85). Obesity is a significant risk factor for VTE and this risk increases with the use of estrogen-containing contraception; compared to normal weight women, women with obesity are estimated to have a 24-fold increase in VTE risk when using combined hormonal contraceptives (85). However, the US Center for Disease Control and Prevention Medical Eligibility Criteria for Contraceptive Use designates combined hormonal contraceptives as risk category 2 (advantages generally outweigh risk) after 6 weeks postpartum in otherwise healthy women with obesity and no cardiovascular risk factors, given the low absolute risk of VTE (85, 97). Women with super-obesity are not specifically addressed. Non-hormonal or progestin-only methods are safe (MEC risk category 1) immediately following delivery in all women (97). Counseling should be had during antenatal care and offering the patient long-acting reversible contraception should be central to the discussion.

Long-acting reversible methods (IUD and/or Nexplanon) are more effective at preventing pregnancy than other methods for women in any BMI category, and ACOG recommends that they be offered to most women as first-line contraception (85). There have been studies finding up to a 50% decrease in etonorgestrel concentrations in women with obesity, although there has not been a decrease in contraceptive efficacy of progesterone implants in those patients with obesity (98).

## Conclusion

Pregnancy for the patient with obesity is more complex than a gravid patient without an elevated BMI, with increasing maternal and fetal risk based on level of obesity. The antepartum, intrapartum, and postpartum needs for these gravidas require greater time, resources, and experience from health care providers. The multitude of health morbidities associated with obesity and super-obesity are beyond the scope of this document; however, obesity in pregnancy has also been associated with accentuation of some of these long-term health risks. Our hope is that this document will offer a comprehensive reference for health care teams when caring for the patient with obesity, and the specific needs for the patient with super obesity, during and after pregnancy.

## Author Contributions

All authors listed have made a substantial, direct, and intellectual contribution to the work and approved it for publication.

## Conflict of Interest

The authors declare that the research was conducted in the absence of any commercial or financial relationships that could be construed as a potential conflict of interest.

## Publisher's Note

All claims expressed in this article are solely those of the authors and do not necessarily represent those of their affiliated organizations, or those of the publisher, the editors and the reviewers. Any product that may be evaluated in this article, or claim that may be made by its manufacturer, is not guaranteed or endorsed by the publisher.

## References

[1] Hales C Carroll M Fryar C Ogden C. Prevalence of Obesity Among Adults Youth: CDC (2017). Available online at: https://www.cdc.gov/nchs/products/databriefs/db288.htm (accessed February 19, 2020).

[2] Ogden C Carroll M Curtin L McDowell M Tabak C Flegal K. Prevalence of overweight and obesity in the United States, 1999–2004. JAMA. (2006) 295:1549–55. 10.1001/jama.295.13.1549 16595758

[3] American College of Obstetricians and Gynecologists. Obesity in pregnancy: ACOG practice bulletin, number 230. Obstet Gynecol. (2021) 137:e128–44. 10.1097/AOG.0000000000004395 34011890

[4] Smid MC Dotters-Katz SK Silver RM Kuller JA. Body mass index 50 kg/m2 and beyond: perioperative care of pregnant women with superobesity undergoing cesarean delivery. Obstet Gynecol Surv. (2017) 72:500–10. 10.1097/OGX.0000000000000469 28817167

[5] ACOG. Clinical Guidelines and Standardization of Practice to Improve Outcomes ACOG.org2019 Replaces CO 629. (2015). Available online at: https://www.acog.org/Clinical-Guidance-and-Publications/Committee-Opinions/Committee-on-Patient-Safety-and-Quality-Improvement/Clinical-Guidelines-and-Standardization-of-Practice-to-Improve-Outcomes (accessed January 8, 2020).

[6] Promotion NCfCDPaH. Defining Adult Overweight Obesity Online: Centers for Disease Control Prevention. (2022). Available online at: https://www.cdc.gov/obesity/basics/adult-defining.html (accessed June 12, 2022).

[7] Group BPA. Bariatric safe patient handling and mobility guidebook: a resource guide for care of persons of size. In: Affairs VH editor. VHA Center for Engineering and Occupational Safety and Health (CEOSH). (2015). p. 1–117.

[8] Poston L Caleyachetty R Cnattingius S Corvalán C Uauy R Herring S. Preconceptional and maternal obesity: epidemiology and health consequences. Lancet Diabetes Endocrinol. (2016) 4:1025–36. 10.1016/S2213-8587(16)30217-0 27743975

[9] O'Brien TE Ray JG Chan WS. Maternal body mass index and the risk of preeclampsia: a systematic overview. Epidemiology. (2003) 14:368–74. 10.1097/01.EDE.0000059921.71494.D1 12859040

[10] Mbah AK Kornosky JL Kristensen S August EM Alio AP Marty PJ. Super-obesity and risk for early and late pre-eclampsia. BJOG. (2010) 117:997–1004. 10.1111/j.1471-0528.2010.02593.x 20482533

[11] Garg J Palaniswamy C Lanier GM. Peripartum cardiomyopathy: definition, incidence, etiopathogenesis, diagnosis, and management. Cardiol Rev. (2015) 23:69–78. 10.1097/CRD.0000000000000038 25111318

[12] Taylor BD Ness RB Olsen J Hougaard DM Skogstrand K Roberts JM. Serum leptin measured in early pregnancy is higher in women with preeclampsia compared with normotensive pregnant women. Hypertension. (2015) 65:594–9. 10.1161/HYPERTENSIONAHA.114.03979 25510827 PMC4326535

[13] Maxwell MH Waks AU Schroth PC Karam M Dornfeld LP. Error in blood-pressure measurement due to incorrect cuff size in obese patients. Lancet. (1982) 2:33–6. 10.1016/S0140-6736(82)91163-1 6123760

[14] Irving G Holden J Stevens R McManus RJ. Which cuff should I use? Indirect blood pressure measurement for the diagnosis of hypertension in patients with obesity: a diagnostic accuracy review. BMJ Open. (2016) 6:e012429. 10.1136/bmjopen-2016-012429 27810973 PMC5129068

[15] Muntner P Shimbo D Carey RM Charleston JB Gaillard T Misra S. Measurement of blood pressure in humans: a scientific statement from the american heart association. Hypertension. (2019) 73:e35–66. 10.1161/HYP.0000000000000087 30827125 PMC11409525

[16] Ferretti A Giampiccolo P Cavalli A Milic-Emili J Tantucci C. Expiratory flow limitation and orthopnea in massively obese subjects. Chest. (2001) 119: 1401–8. 10.1378/chest.119.5.1401 11348945

[17] Sood A. Altered resting and exercise respiratory physiology in obesity. Clin Chest Med. (2009) 30:445–54. 10.1016/j.ccm.2009.05.003 19700043 PMC2765111

[18] Stahl J Malhotra S. Obesity Surgery Indications And Contraindications. Treasure Island: StatPearls Publishing (2019). 30020657

[19] Lopez PP Stefan B Schulman CI Byers PM. Prevalence of sleep apnea in morbidly obese patients who presented for weight loss surgery evaluation: more evidence for routine screening for obstructive sleep apnea before weight loss surgery. Am Surg. (2008) 74:834–8. 10.1177/000313480807400914 18807673

[20] Nagappa M Liao P Wong J Auckley D Ramachandran SK Memtsoudis S. Validation of the STOP-bang questionnaire as a screening tool for obstructive sleep apnea among different populations: a systematic review and meta-analysis. PLoS ONE. (2015) 10:e0143697. 10.1371/journal.pone.0143697 26658438 PMC4678295

[21] Dominguez JE Krystal AD Habib AS. Obstructive sleep apnea in pregnant women: a review of pregnancy outcomes and an approach to management. Anesth Analg. (2018) 127:1167–77. 10.1213/ANE.0000000000003335 29649034 PMC6733415

[22] Louis JM Mogos MF Salemi JL Redline S Salihu HM. Obstructive sleep apnea and severe maternal-infant morbidity/mortality in the United States, 1998–2009. Sleep. (2014) 37:843–9. 10.5665/sleep.3644 24790262 PMC3985102

[23] Marchi J Berg M Dencker A Olander EK Begley C. Risks associated with obesity in pregnancy, for the mother and baby: a systematic review of reviews. Obes Rev. (2015) 16:621–38. 10.1111/obr.12288 26016557

[24] Simon R. Catherine R-P Nicholas C. H Selby P John W. Vitamin D in Pregnancy. Oxford, UK: Royal College of Obstetricians and Gynaecologists (2014).

[25] Bodnar LM Catov JM Roberts JM Simhan HN. Prepregnancy obesity predicts poor vitamin D status in mothers and their neonates. J Nutr. (2007) 137:2437–42. 10.1093/jn/137.11.2437 17951482 PMC2556251

[26] Force UPST. Screening for gestational diabetes: US preventive services task force recommendation statement. JAMA. (2021) 326:531–8. 10.1001/jama.2021.11922 34374716

[27] Ge GM Leung MTY Man KKC Leung WC Ip P Li GHY. Maternal thyroid dysfunction during pregnancy and the risk of adverse outcomes in the offspring: a systematic review and meta-analysis. J Clin Endocrinol Metab. (2020) 105:3821–41. 10.1210/clinem/dgaa555 32810262

[28] Alexander EK Pearce EN Brent GA Brown RS Chen H Dosiou C. (2017) Guidelines of the american thyroid association for the diagnosis and management of thyroid disease during pregnancy and the postpartum. Thyroid. (2017) 27:315–89. 10.1089/thy.2016.0457 28056690

[29] American College of Obstetricians and Gynecologists. Thyroid disease in pregnancy: ACOG practice bulletin summary, number 223. Obstet Gynecol. (2020) 135:1496–9. 10.1097/AOG.0000000000003894 32443078

[30] Lazarus JH Bestwick JP Channon S Paradice R Maina A Rees R. Antenatal thyroid screening and childhood cognitive function. N Engl J Med. (2012) 366:493–501. 10.1056/NEJMoa1106104 22316443 PMC3814060

[31] Casey BM Thom EA Peaceman AM Varner MW Sorokin Y Hirtz DG. Treatment of subclinical hypothyroidism or hypothyroxinemia in pregnancy. N Engl J Med. (2017) 376:815–25. 10.1056/NEJMoa1606205 28249134 PMC5605129

[32] Guidelines IoMUaNRCUCtRIPW. Weight Gain During Pregnancy: Reexamining the Guidelines (2009). Washington, DC: National Academies Press. 20669500

[33] Hinkle SN Sharma AJ Dietz PM. Gestational weight gain in obese mothers and associations with fetal growth. Am J Clin Nutr. (2010) 92:644–51. 10.3945/ajcn.2010.29726 20631201

[34] Catalano PM Mele L Landon MB Ramin SM Reddy UM Casey B. Inadequate weight gain in overweight and obese pregnant women: what is the effect on fetal growth? Am J Obstet Gynecol. (2014) 211:137.e1–7. 10.1016/j.ajog.2014.02.004 24530820 PMC4117705

[35] Kapadia MZ Park CK Beyene J Giglia L Maxwell C McDonald SD. Weight loss instead of weight gain within the guidelines in obese women during pregnancy: a systematic review and meta-analyses of maternal and infant outcomes. PLoS ONE. (2015) 10:e0132650. 10.1371/journal.pone.0132650 26196130 PMC4509670

[36] Force UPST. Behavioral counseling interventions for healthy weight and weight gain in pregnancy: US preventive services task force recommendation statement. JAMA. (2021) 325:2087–93. 10.1001/jama.2021.6949 34032823

[37] Lashen H Fear K Sturdee DW. Obesity is associated with increased risk of first trimester and recurrent miscarriage: matched case-control study. Hum Reprod. (2004) 19:1644–6. 10.1093/humrep/deh277 15142995

[38] Stothard KJ Tennant PW Bell R Rankin J. Maternal overweight and obesity and the risk of congenital anomalies: a systematic review and meta-analysis. JAMA. (2009) 301:636–50. 10.1001/jama.2009.113 19211471

[39] Zwink N Jenetzky E Brenner H. Parental risk factors and anorectal malformations: systematic review and meta-analysis. Orphanet J Rare Dis. (2011) 6:25. 10.1186/1750-1172-6-25 21586115 PMC3121580

[40] Van Lieshout RJ Taylor VH Boyle MH. Pre-pregnancy and pregnancy obesity and neurodevelopmental outcomes in offspring: a systematic review. Obesity Rev. (2011) 12:e548–e59. 10.1111/j.1467-789X.2010.00850.x 21414129

[41] American Institute of Ultrasound in Medicine. AIUM practice parameter for the performance of detailed second- and third-trimester diagnostic obstetric ultrasound examinations. J Ultrasound Med. (2019) 38:3093–100. 10.1002/jum.15163 31736130

[42] Maxwell C Glanc P. Imaging and obesity: a perspective during pregnancy. Am J Roentgenol. (2011) 196:311–9. 10.2214/AJR.10.5849 21257881

[43] Tanner LD Brock And C Chauhan SP. Severity of fetal growth restriction stratified according to maternal obesity. J Matern Fetal Neonatal Med. (2020):1–5. 10.1080/14767058.2020.1773427 32482116

[44] Pugh SJ Ortega-Villa AM Grobman W Newman RB Owen J Wing DA. Estimating gestational age at birth from fundal height and additional anthropometrics: a prospective cohort study. BJOG. (2018) 125:1397–404. 10.1111/1471-0528.15179 29473290 PMC6107439

[45] Aune D Saugstad OD Henriksen T Tonstad S. Maternal body mass index and the risk of fetal death, stillbirth, and infant death: a systematic review and meta-analysis. JAMA. (2014) 311:1536–46. 10.1001/jama.2014.2269 24737366

[46] Yao R Ananth CV Park BY Pereira L Plante LA Consortium PR. Obesity and the risk of stillbirth: a population-based cohort study. Am J Obstet Gynecol. (2014) 210:457.e1–9. 10.1016/j.ajog.2014.01.044 24674712

[47] Metz TD Berry RS Fretts RC Reddy UM Turrentine MA. Obstetric care consensus #10: management of stillbirth: (replaces practice bulletin number 102, March 2009). Am J Obstet Gynecol. (2020) 222:B2–20. 10.1016/j.ajog.2020.01.017 32004519

[48] Bodnar LM Parks WT Perkins K Pugh SJ Platt RW Feghali M. Maternal prepregnancy obesity and cause-specific stillbirth. Am J Clin Nutr. (2015) 102:858–64. 10.3945/ajcn.115.112250 26310539 PMC4588742

[49] Gould JB Mayo J Shaw GM Stevenson DK. Medicine MoDPRCaSUSo Swedish and American studies show that initiatives to decrease maternal obesity could play a key role in reducing preterm birth. Acta Paediatr. (2014) 103:586–91. 10.1111/apa.12616 24575829

[50] Tonidandel A Booth J D'Angelo R Harris L Tonidandel S. Anesthetic and obstetric outcomes in morbidly obese parturients: a 20-year follow-up retrospective cohort study. Int J Obstet Anesth. (2014) 23:357–64. 10.1016/j.ijoa.2014.05.004 25201313

[51] Lucas DN. The 30 minute decision to delivery time is unrealistic in morbidly obese women. Int J Obstet Anesth. (2010) 19:431–5. 10.1016/j.ijoa.2010.07.020 20833528

[52] Vallejo MC. Anesthetic management of the morbidly obese parturient. Curr Opin Anaesthesiol. (2007) 20:175–80. 10.1097/ACO.0b013e328014646b 17479016

[53] Eley VA Christensen R Guy L Dodd B. Perioperative blood pressure monitoring in patients with obesity. Anesth Analg. (2019) 128:484–91. 10.1213/ANE.0000000000003647 30059399

[54] Subramaniam A Jauk VC Goss AR Alvarez MD Reese C Edwards RK. Mode of delivery in women with class III obesity: planned cesarean compared with induction of labor. Am J Obstet Gynecol. (2014) 211:e1–9. 10.1016/j.ajog.2014.06.045 24956550

[55] Norman SM Tuuli MG Odibo AO Caughey AB Roehl KA Cahill AG. The effects of obesity on the first stage of labor. Obstet Gynecol. (2012) 120:130–5. 10.1097/AOG.0b013e318259589c 22914401 PMC4494673

[56] Frolova AI Raghuraman N Stout MJ Tuuli MG Macones GA Cahill AG. Obesity, second stage duration, and labor outcomes in nulliparous women. Am J Perinatol. (2019). 10.1055/s-0039-1697586 31563134 PMC8081034

[57] Davidoff A Reuter K Karellas A Baker SP Raptopoulos V. Maternal umbilicus: ultrasound window to the gravid uterus. J Clin Ultrasound. (1994) 22:263–7. 10.1002/jcu.1870220409 8006186

[58] James DC Maher MA. Caring for the extremely obese woman during pregnancy and birth. MCN Am. J. Maternal/Child Nursing. (2009) 34:24–30. 10.1097/01.NMC.0000343862.72237.62 19104316

[59] American College of Obstetricians and Gynecologists. ACOG Practice Bulletin No. 106: Intrapartum fetal heart rate monitoring: nomenclature, interpretation, and general management principles. Obstet Gynecol. (2009) 114:192–202. 10.1097/AOG.0b013e3181aef106 19546798

[60] Monson M Heuser C Einerson BD Esplin I Snow G Varner M. Evaluation of an external fetal electrocardiogram monitoring system: a randomized controlled trial. Am J Obstet Gynecol. (2020) 223:244.e1–.e12. 10.1016/j.ajog.2020.02.012 32087146 PMC8842851

[61] Smid MC. Kearney, MS, Stamilio DM. Extreme obesity and postcesarean wound complications in the maternal-fetal medicine unit cesarean registry. Am J Perinatol. (2015) 32:1336–41. 10.1055/s-0035-1564883 26489063

[62] Conner SN Verticchio JC Tuuli MG Odibo AO Macones GA Cahill AG. Maternal obesity and risk of postcesarean wound complications. Am J Perinatol. (2014) 31:299–304. 10.1055/s-0033-1348402 23765707 PMC3796045

[63] Pevzner L Swank M Krepel C Wing DA Chan K Edmiston CE. Effects of maternal obesity on tissue concentrations of prophylactic cefazolin during cesarean delivery. Obstet Gynecol. (2011) 117:877–82. 10.1097/AOG.0b013e31820b95e4 21422859

[64] Swank ML Wing DA Nicolau DP McNulty JA. Increased 3-gram cefazolin dosing for cesarean delivery prophylaxis in obese women. Am J Obstet Gynecol. (2015) 213:415.e1–8. 10.1016/j.ajog.2015.05.030 26003059

[65] Haas DM Morgan S Contreras K Kimball S. Vaginal preparation with antiseptic solution before cesarean section for preventing postoperative infections. Cochrane Database Syst Rev. (2020) 4:CD007892. 10.1002/14651858.CD007892.pub7 32335895 PMC7195184

[66] Denison F Aedla N Keag O Hor K Reynolds R Milne A. Care of women with obesity in pregnancy. BJOG. (2018). 10.1111/1471-0528.15386 30465332

[67] Bates SM Middeldorp S Rodger M James AH Greer I. Guidance for the treatment and prevention of obstetric-associated venous thromboembolism. J Thromb Thrombolysis. (2016) 41:92–128. 10.1007/s11239-015-1309-0 26780741 PMC4715853

[68] Wall PD Deucy EE Glantz JC Pressman EK. Vertical skin incisions and wound complications in the obese parturient. Obstet Gynecol. (2003) 102:952–6. 10.1016/S0029-7844(03)00861-5 14672469

[69] Thornburg LL. Antepartum obstetrical complications associated with obesity. Semin Perinatol. (2011) 35:317–23. 10.1053/j.semperi.2011.05.015 22108080

[70] Sutton AL Sanders LB Subramaniam A Jauk VC Edwards RK. Abdominal incision selection for cesarean delivery of women with class III obesity. Am J Perinatol. (2016) 33:547–51. 10.1055/s-0035-1570339 26692204

[71] McLean M Hines R Polinkovsky M Stuebe A Thorp J Strauss R. Type of skin incision and wound complications in the obese parturient. Am J Perinatol. (2012) 29:301–6. 10.1055/s-0031-1295637 22105439

[72] Walton RB Shnaekel KL Ounpraseuth ST Napolitano PG Magann EF. High transverse skin incisions may reduce wound complications in obese women having cesarean sections: a pilot study. J Matern Fetal Neonatal Med. (2019) 32:781–5. 10.1080/14767058.2017.1391780 29020834

[73] Naumann RW Hauth JC Owen J Hodgkins PM Lincoln T. Subcutaneous tissue approximation in relation to wound disruption after cesarean delivery in obese women. Obstet Gynecol. (1995) 85:412–6. 10.1016/0029-7844(94)00427-F 7862382

[74] Zaki MN Wing DA McNulty JA. Comparison of staples vs subcuticular suture in class III obese women undergoing cesarean: a randomized controlled trial. Am J Obstet Gynecol. (2018) 218:451.e1–.e8. 10.1016/j.ajog.2018.02.011 29474843

[75] Yu L Kronen RJ Simon LE Stoll CRT Colditz GA Tuuli MG. Prophylactic negative-pressure wound therapy after cesarean is associated with reduced risk of surgical site infection: a systematic review and meta-analysis. Am J Obstet Gynecol. (2018) 218:200–10.e1. 10.1016/j.ajog.2017.09.017 28951263 PMC5807120

[76] Tuuli MG Liu J Tita ATN Longo S Trudell A Carter EB. Effect of prophylactic negative pressure wound therapy vs standard wound dressing on surgical-site infection in obese women after cesarean delivery: a randomized clinical trial. JAMA. (2020) 324:1180–9. 10.1001/jama.2020.13361 32960242 PMC7509615

[77] Mhyre JM D'Oria R Hameed AB Lappen JR Holley SL Hunter SK. The maternal early warning criteria: a proposal from the national partnership for maternal safety. Obstet Gynecol. (2014) 124:782–6. 10.1097/AOG.0000000000000480 25198266

[78] Camden S. Obesity: an emerging concern for patients and nurses. Online J Issues Nurs. (2009) 14:1–8. 10.3912/OJIN.Vol14No1Man01

[79] Kim C Newton KM Knopp RH. Gestational diabetes and the incidence of type 2 diabetes: a systematic review. Diabetes Care. (2002) 25:1862–8. 10.2337/diacare.25.10.1862 12351492

[80] National Collaborating Centre for Women's Children's Health (UK). Hypertension in Pregnancy: The Management of Hypertensive Disorders During Pregnancy. London, UK: RCOG Press (2010). 22220321

[81] Page KA Romero A Buchanan TA Xiang AH. Gestational diabetes mellitus, maternal obesity, and adiposity in offspring. J Pediatr. (2014) 164:807–10. 10.1016/j.jpeds.2013.11.063 24388326 PMC3962700

[82] Nijs H Benhalima K. Gestational diabetes mellitus and the long-term risk for glucose intolerance and overweight in the offspring: a narrative review. J Clin Med. (2020) 9. 10.3390/jcm9020599 32098435 PMC7074239

[83] Blomberg M. Maternal obesity and risk of postpartum hemorrhage. Obstet. Gynecol. (2011) 118. 10.1097/AOG.0b013e31822a6c59 21860284

[84] Butwick AJ Abreo A Bateman BT Lee HC El-Sayed YY Stephansson O. Effect of maternal body mass index on postpartum hemorrhage. Anesthesiology. (2018) 128:774–83. 10.1097/ALN.0000000000002082 29346134 PMC5849500

[85] Martin A Krishna I Ellis J Paccione R Badell M. Super obesity in pregnancy: difficulties in clinical management. J Perinatol. (2014) 34:495–502. 10.1038/jp.2014.4 24503915

[86] American College of Obstetricians and Gynecologists' Committee on Practice Bulletins—Obstetrics. ACOG Practice Bulletin No 196: Thromboembolism in Pregnancy. Obstet Gynecol. (2018) 132:e1–e17. 10.1097/AOG.0000000000002706 29939938

[87] Gould MK Garcia DA Wren SM Karanicolas PJ Arcelus JI Heit JA. Prevention of VTE in nonorthopedic surgical patients: antithrombotic therapy and prevention of thrombosis, 9th ed: american college of chest physicians evidence-based clinical practice guidelines. Chest. (2012) 141:e227S–e77S. 10.1378/chest.11-2297 22315263 PMC3278061

[88] Bahl V Hu HM Henke PK Wakefield TW Campbell DA Jr Caprini JA. A validation study of a retrospective venous thromboembolism risk scoring method. Ann Surg. (2010) 251:344–50. 10.1097/SLA.0b013e3181b7fca6 19779324

[89] D'Alton ME Friedman AM Smiley RM Montgomery DM Paidas MJ D'Oria R. National partnership for maternal safety: consensus bundle on venous thromboembolism. Anesthesia Analgesia. (2016) 123. 10.1213/ANE.0000000000001569 27636577

[90] Barbar S Noventa F Rossetto V Ferrari A Brandolin B Perlati M. A risk assessment model for the identification of hospitalized medical patients at risk for venous thromboembolism: the Padua Prediction Score. J Thromb Haemost. (2010) 8:2450–7. 10.1111/j.1538-7836.2010.04044.x 20738765

[91] Westerfield KL Koenig K Oh R. Breastfeeding: common questions and answers. Am Fam Physician. (2018) 98:368–73. 30215910

[92] Amir LH Donath S. A systematic review of maternal obesity and breastfeeding intention, initiation and duration. BMC Pregnancy Childbirth. (2007) 7:9. 10.1186/1471-2393-7-9 17608952 PMC1937008

[93] Chapman DJ Pérez-Escamilla R. Identification of risk factors for delayed onset of lactation. J Am Diet Assoc. (1999) 99:450-4. 10.1016/S0002-8223(99)00109-1 10207398

[94] Rasmussen KM Kjolhede CL. Prepregnant overweight and obesity diminish the prolactin response to suckling in the first week postpartum. Pediatrics. (2004) 113:e465–71. 10.1542/peds.113.5.e465 15121990

[95] Demirci J Schmella M Glasser M Bodnar L Himes KP. Delayed Lactogenesis II and potential utility of antenatal milk expression in women developing late-onset preeclampsia: a case series. BMC Pregnancy Childbirth. (2018) 18:68. 10.1186/s12884-018-1693-5 29544467 PMC5855986

[96] Simmons KB Edelman AB. Hormonal contraception and obesity. Fertil Steril. (2016) 106:1282–8. 10.1016/j.fertnstert.2016.07.1094 27565257

[97] Curtis KM Tepper NK Jatlaoui TC Berry-Bibee E Horton LG Zapata LB. U.S. medical eligibility criteria for contraceptive use, 2016. MMWR Recomm Rep. (2016) 65:1–103. 10.15585/mmwr.rr6503a1 27467196

[98] Xu H Wade JA Peipert JF Zhao Q Madden T Secura GM. Contraceptive failure rates of etonogestrel subdermal implants in overweight and obese women. Obstet Gynecol. (2012) 120:21–6. 10.1097/AOG.0b013e318259565a 22678035 PMC4043143

