# Supplementary material for: Pregnancy Care for Patients With Super Morbid Obesity
Source: Front Pediatr. 2022 Jul 19;10:839377. doi: 10.3389/fped.2022.839377 (PMC9343711; doi:10.3389/fped.2022.839377)
Supplement: Supplementary file 1 [file Table_1.DOCX]

# CHECKLIST

## Antepartum

- Extensively counsel patients about potential medical complications of obesity; consider MFM consultation with BMI >40 kg/m^2^, recommend MFM consultation with BMI >50 kg/m^2^
- First trimester dating ultrasound
- Recommend weight gain of 11-20 pounds for BMI ≥30 kg/m^2^
- Consultation and follow-up with a nutritionist
- Additional first trimester labs: diabetes screening with HbgA1c or one-hour glucose tolerance test, vitamin D level, thyroid function tests (if BMI of >40 kg/m^2^)
- Baseline preeclampsia lab assessment: AST, ALT, creatinine, 24 hour urine collection
- Screening transthoracic echo and ECG in patients with BMI >50 kg/m^2^
- Screen for sleep apnea and refer to sleep specialist for OSA symptoms
- Counsel on the limitations of genetic screening, particularly the risk of indeterminate cell free DNA screening. If obtaining serum analyte screening, precise and up-to-date weights are required.
- Detailed anatomy ultrasound at 20-22 weeks in the absence of an indication for an early anatomic survey; counsel patient regarding limitations of ultrasound in setting of maternal habitus.
- Serial growth ultrasound secondary to inability to measure fundal heights
- Anesthesia consultation if BMI ≥40 kg/m^2^
- Antenatal testing as dictate by comorbid conditions OR weekly at:
  - 37 weeks, if pre-pregnancy BMI 35-39 kg/m^2^
  - 34 weeks, if pre-pregnancy BMI was >40 kg/m^2^
- Delivery timing for usual indications; consider 39 week induction
- Lactation planning: prenatal lactation consult, antenatal hand expression
- Contraceptive counseling

**Intrapartum**

- Early Anesthesia evaluation on admission to L&D
- Secure IV access
- Clear intrapartum fetal monitoring plans; consider early amniotomy for placement of internal monitors
- No change to GBS antibiotic prophylaxis
- Consider allowing for an extended first stage of labor prior to diagnosing arrest of labor

***OR PLANNING***

- Adequate personnel in OR to help with positioning
- Availability of ultrasound to assist with FHR monitoring
- Careful assessment of abdomen and decision regarding incision type; availability of subcutaneous retraction devices
- Communication with Anesthesia regarding possible thoracic epidural placement if supra-umbilical incision.
- Sufficient abdominal prep supplies
- Increase preoperative cefazolin dosing to 3 gm IV in women with weight >120 kg or a BMI ≥40 kg/m^2^ (no changes to gentamycin/clindamycin). Vancomycin- increase dosing based on weight class.

**Postpartum**

- Early ambulation and lactation support
- Recommend pharmacologic thromboprophylaxis following:
  - Vaginal delivery in patients with BMI ≥30kg/m^2^ AND any additional risk factor
  - Cesarean delivery in all patients, regardless of BMI.
- Contraceptive choice
- Referral/transition of care to primary care provider with detailed report of pregnancy comorbidities; close monitoring of blood pressure in the postpartum period and testing for underlying diabetes as indicated.
